# 2835. Utilization of Clinical-Grade Metagenomics in Urinary Tract Infection Diagnostics: Improving High-Risk Patients’ Outcomes with a Genomic-Based Assay

**DOI:** 10.1093/ofid/ofad500.2445

**Published:** 2023-11-27

**Authors:** Eduardo J Morfa Romero, Michael Augenbraun, Tiara Rivera, Sol Rey, Xavier O Jirau Serrano, John C Papciak, Suraj S Patel, Bridget Harrison, Anna Plourde, Caitlin Otto, Chris Mason, Heather L Wells, David C Danko, Niamh B O’Hara, Mara Couto-Rodriguez, Dorottya Nagy-Szakal

**Affiliations:** SUNY Downstate Health Sciences University, Brooklyn, New York; SUNY Downstate Medical University, Brooklyn, NY; Biotia, Brooklyn, New York; Biotia Inc., Brooklyn, New York; Biotia Inc., Brooklyn, New York; Biotia Inc., Brooklyn, New York; Biotia Inc., New York, NY, USA, Elmhurst, New York; Biotia, Brooklyn, New York; SUNY Downstate Medical Center, Brooklyn, New York; Biotia Inc., Brooklyn, New York; Biotia, Brooklyn, New York; Biotia, Brooklyn, New York; Biotia, Brooklyn, New York; Biotia Inc., Brooklyn, New York; Biotia Inc., Brooklyn, New York; Biotia Inc., Brooklyn, New York

## Abstract

**Background:**

Immunosuppressed patients and those experiencing complicated UTIs are more susceptible to misdiagnosis by outdated standard of care (SOC) diagnostics; resulting in ineffective treatment and risk of severe complications. Shotgun metagenomic sequencing can act as a highly accurate tool in profiling urogenital pathogens in high-risk patients. We assessed the performance of a genomic-based urine assay in patients where the SOC failed to identify pathogens and compiled clinical metadata from medical records to evaluate if antimicrobial stewardship could be improved.

**Methods:**

Culture negative (no growth or < 100k CFU/mL) urine specimens (n=140) were collected from patients with pyuria and UTI symptoms then processed with the BIOTIA-ID Urine NGS Assay including DNA extraction, library preparation, Illumina NextSeq sequencing and analysis by BIOTIA-DX to identify urogenital pathogens. Findings were correlated with clinical metadata and administered antimicrobial therapies (Advarra Pro00038083, IRBNet 1950413-1).

**Results:**

Our assay yielded an average of 2M microbial reads per sample with 98.6% passing laboratory QC. Microbial species were detected in 82.6% of the culture-negative specimens, of which 36.8% were single organisms and 45.6% were polymicrobial infections. Different pathogen profiles were distinguished based on prior antibiotic use, catheterization, or specific comorbidities (kidney transplant, cancer and prostatic hyperplasia). 50% of septic patients showed single organism infections, while cancer patients had higher incidence of polymicrobial infections (52%). Higher prevalence of fungal infections (20%) were observed in catheterized patients, and those with prior antibiotic therapy within 30 days. Retrospective evaluation revealed 63% prescribed antimicrobials would not target the organisms detected. Further, 57% of the cases could have potentially de-escalated their treatment.

**Conclusion:**

High-risk patients frequently harbor atypical pathogens leading to inaccurate SOC testing and ineffective therapeutics. Genomic-based diagnostics may improve accuracy of urogenital pathogen detection and offer physicians precision therapeutics with better health outcomes while enhancing antimicrobial stewardship.

**Disclosures:**

**Tiara Rivera, B.S.**, Biotia Inc.: Research Staff **Sol Rey, BS**, Biotia Inc: Researcher-Staff **Xavier O. Jirau Serrano, MS**, Biotia Inc: Research and development staff **John C. Papciak, BS**, Biotia Inc.: Staff **Suraj S. Patel, n/a**, Biotia Inc.: staff **Bridget Harrison, B.A.**, Biotia Inc.: Clinical Program Manager- Staff **Caitlin Otto, PhD, D(ABMM)**, Biotia Inc.: Staff **Chris Mason, Ph.D.**, Biotia: Board Member|Biotia: Stocks/Bonds **Heather L. Wells, MPH, PhD Candidate**, Biotia, Inc.: Employee **David C. Danko, Ph.D.**, Biotia Inc: Stocks/Bonds **Niamh B. O'Hara, PhD**, Biotia: Board Member|Biotia: 2 patents|Biotia: Ownership Interest|Biotia: Stocks/Bonds **Mara Couto-Rodriguez, MS**, Biotia Inc: Employee **Dorottya Nagy-Szakal, MD PhD**, Biotia Inc.: Employee|Biotia Inc.: Stocks/Bonds

